# Mental health professionals’ expectations and efforts to include employment for people with moderate to severe mental illness in treatment settings

**DOI:** 10.1186/s12888-023-04568-4

**Published:** 2023-01-31

**Authors:** Joakim Finne, Karin Holt

**Affiliations:** 1 Work Research Institute, Oslo Metropolitan University, Oslo, Norway; 2 Regional Center for Violence, Traumatic Stress, and Suicide Prevention, Oslo, Norway; 3 Division of Mental Health Services, Akershus University Hospital, 1478 Lørenskog, Norway

**Keywords:** Psychiatry, Mental health, Occupational mental health, Rehabilitation, Evidence-based practice

## Abstract

**Background:**

Research suggests that employment is an important factor for recovery and improved quality of life for people with mental illnesses. Mental health professionals often serve as gatekeepers for employment interventions, yet little is known about their expectations about employment for people with mental illness in Norway. The purpose of this study is to examine mental health professionals’ expectations and efforts to include employment for people with moderate to severe mental illness in treatment settings.

**Methods:**

Two hundred seven mental health professionals were recruited from municipal mental health services, specialized clinics, social media, and professionals’ networks across Norway. Participants completed a survey package comprising demographic questions, current practices and a revised version of the self-reported measure Expectations for the Employability of People with Serious Mental Illness (EESMI), a validated measure consisting of three subscales.

**Results:**

Results suggested overall favorable expectations of employment for people with moderate to severe mental illness. Analyses revealed that patients participating more frequently in collaborative meetings predicted more favorable expectations about employment among mental health professionals compared to less frequent meetings. In addition, findings suggest that psychiatrist hold more negative expectations about employment in comparison to the other educational groups. Lastly, more than half of mental health professionals reported that they have integrated discussions about employment, and routines to address work-oriented activity in consultations with patients, however, there are substantial variations in routines for addressing work or work-oriented activity as a topic in consultations with patients.

**Conclusions:**

These results suggest that efforts are being made to integrate employment in treatment settings for people with mental illness in Norway; however, more work is needed to remove barriers and facilitate evidence-based approaches.

## Background

On the OECD Better Life Index, Norway ranks top in personal security, above average in subjective well-being, jobs and earnings, income and wealth, education and skills, housing, work-life balance, civic engagement, social connections, and health status [1]. Yet, reports from the National Institute of Public Health reveal that mental disorders are widespread in the Norwegian population which contribute to a significant decline in health. It is estimated that approximately half of the Norwegian population will be affected by a mental disorder during their lifetime, and approximately one third within a year [2]. Research suggests that there is a strong connection between unemployment among young people and mental health in high-income countries. Unemployment is often associated with increased mental health problems, depression, and anxiety disorders, and is often fueled by stigma related to mental disorders [3, 4]. For those with mental illnesses, work is considered a contributing factor to a feeling of normality, acceptance, belonging and fulfillment of norms and values [5, 6], given that there are favorable workplace conditions [7].

Common employment interventions in Norway include Individual Placement and Support (IPS), which has demonstrated promising results for supporting people with significant disabilities to reach paid employment [8]. The model is arguably one of the most robust employment interventions for people with mental health disorders [9–11]. Studies on IPS have demonstrated that subjects in IPS, compared to usual treatment conditions, have better vocational outcomes, job tenure, job length, global functioning, improved mental health and an increased reason to expect a possible improvement for quality of life for at least some settings [11]. These findings advocate the significance employment can have for recovery and improved quality of life for people with mental illnesses. Yet, OECD found that people with severe and common mental disorders are seven and three times more likely respectively to be unemployed compared to people with no mental disorders [12]. There are several barriers identified in research literature for workforce discrimination against people with mental illness.

For instance, stigma in the workplace, inadequate training opportunities, and lack of ongoing integrated funding for programming are central barriers for employment [13]. Other barriers include cost of employment interventions [14] and negative attitudes and misconstructions among mental health professionals—for instance, the idea that the patient must be symptom free before starting vocational training [15] and skepticism towards the feasibility of work for people with mental illness [16]. Research suggests that employee reluctance often includes concerns related to social skills, fear of long-term absence, workload, trust, and the need for additional supervision [17, 18].

Among mental health providers, Kochański et al. [19] surveyed 232 psychiatrists on their attitudes towards people with mental illnesses. Compared to the general population, 43% of the psychiatrists stated that mental illness significantly decreases the ability of regular work, and 13.5% reported that it decreases the ability to work in a team. These results are echoed in the study by Roets et al. who explored the experiences of five individuals with long term mental health problems searching for employment. The authors argue that people with mental health problems often face prejudice and stereotypes among mental health providers, such as the belief that the patient should be recovered prior to seeking out employment opportunities [20]. Low expectations of employment for people with mental illness were also found in the study by Costa et al. who examined 1306 mental health providers views towards employment and recovery among people with serious mental illness. The authors found that mental health providers rated employment and finances as the least important factor for recovery for people with serious mental illness [21].

Previous research has demonstrated that mental health providers' beliefs, support and involvement in the patient’s employment goals have proven to be important factors when comparing high and low-performing employment programs for people with mental illness [22]. Examining how mental health professionals integrate employment is important as they often serve as gatekeepers for employment interventions through collaborative efforts with employers or social services. For example, mental health professionals in Norway play an integral part in referring patients to the employment interventions such as IPS. Better understandings of employment practices can contribute to building evidence-based employment practices by emphasizing the best available evidence, patients’ preference, and the professional’s clinical expertise [23], by integrating the concept of employment in treatment practices.

### The Norwegian context

The Norwegian mental health system is organized at the municipal and state level. The municipalities run primary healthcare, which includes general practitioners, team-based primary mental health, and substance abuse care [24]. The specialist services which are organized at the state level solve tasks that require competence and resources beyond what is covered in the municipal healthcare services. These include specialized mental health clinics, specialized drug addiction and treatment clinics, and hospital wards with specialized units. Mental health care at the state level interacts with the municipalities and social services as well as other departments in the specialist health service.

The Norwegian social services are organized at the municipality and state level and consist of Labor and Welfare Services and municipal agencies. Social services are responsible for some employment schemes and various employment intervention programs, some of which are tailored to people with mental illness, such as IPS for people with moderate to severe mental illness. While collaboration agreements have been made between social services and the health trusts in most counties in Norway, there are variations in how well the agreements are anchored, and to what extent the content of the collaboration is concretized [25].

### Aim of the study

The aim of this study is to examine mental health professionals’ expectations and efforts to include employment for people with moderate to severe mental illness. This includes first examining mental health professionals’ expectations about employment through EESMI (Expectations for the Employability of People with Serious Mental Illness), and then looking at aspects of how employment is integrated in current practices across sectors and professions.

## Methods

### Data collection procedure

The data were collected from mental health professionals working in primary and specialist health services across Norway between August 2021 and October 2021. The authors used a survey tool with a high degree of security and privacy (Nettskjema), to collect sensitive data. The selection of mental health professionals was conducted through a combination of purposive sampling and snowball sampling to get a variety of gender, geographical backgrounds, professional backgrounds, and fields of practice. The survey was sent to a common e-mail list for primary and specialist health services across Norway and posted in Facebook groups designated for mental health professionals. Snowball sampling techniques were used by sending an electronic questionnaire to mental health professionals in the second author’s professional and clinical networks, who were asked to forward the email to their professional network. The first page of the survey included information regarding study participation, anonymity, privacy, security, and data management.

### Measures

This survey was developed to gather data on employment and collaborative practices among Norwegian mental health professionals. The survey included 49 questions about educational background, professional training, tenure, employment, collaborative practices, and demographic questions. To determine how employment is discussed and integrated into local routines among mental health professionals, participants were asked the following questions used in the analysis. “How often are patients’ ability to work/employment opportunities, work-oriented activity or collaboration with social services a topic in staff meetings the unit?” and “Does the unit have routines for mental health professionals to address work or work-oriented activity as a topic in consultations with patients (for example, conversation tools or treatment plan?”.

#### Measuring expectations about employment

In this study we utilized the Expectations for the Employability of People with Serious Mental Illness (EESMI) developed by Abraham et al. [26]. EESMI is a psychometrically sound measure that consists of 23 items that measure mental health professionals’ expectations about client employment. The measure covers three themes that have strong internal consistency on each subscale, respectively measuring case manager expectations of (a) benefits of work which reflect the expectations that people with serious mental illness are capable of working and that employment has a number of benefits for people with serious mental illness, (b) demands of the worker role which reflects the individual’s expectation that people with moderate to severe mental illness fit social roles at work, that employers are willing to hire them, that co-workers wish to socialize with them, and that they are able to do their fair share of work and lastly (c) motivation to work reflects expectations that individuals with moderate to severe mental illness are motivated to pursue and maintain employment.

We adapted the measure from a 3- point scale to a 7-point scale to increase granularity (Alwin & Krosnick, 1991). In addition, the wording of the questionnaire was adapted to assess expectations for the employability of people with moderate to severe mental illness, to account for local wording and practices in the Norwegian mental health system. The Norwegian translation of EESMI was conducted by a psychology student and back translated by the first author. The translation followed guidelines of cross-cultural translation, adaptation, and validation of instruments [27]. Following the procedure by Abraham et al. [26], 11 items were reverse scored so that higher scores indicate more favorable expectations of employment.

#### Background variables

The dummy variable “Master’s degree” used in the regression analysis, comprising those with a higher education than a bachelor’s degree, with and without continuing education. Participants who had more than 10 years of tenure were coded as “1” and the rest were coded as “0”. The variable “Interaction with social services” comprised those who “often,” “very often” or “always” interact with social services during the course of treatment. The participants were asked the question “Is the patient attending meetings with external service providers (service providers outside of the participants department such as social services, child welfare services, various municipal health services), when the focus of the meeting is about the patient him-/herself?”. A dummy variable labelled “Patient participation” comprised those who participate “often,” “very often” and “always.

### Statistical analysis

We used version 27.0 of SPSS to analyze our data. After data cleaning we calculated descriptive statistics, mean scores and standard deviations for background variables, participant characteristics, and EESMI. Crosstabulation was used to understand the relationship between some of the background variables. A one-way ANOVA and multiple comparisons were used to examine the relationships between the four largest educational groups and expectations about employment. Lastly, linear regression was used to predict and explain the effects of the independent variables on the dependent variables.

## Results

### Description of participants

The participants in this study comprised 207 mental health professionals across Norway. As seen in Table 1, the majority (84.1%) of the participants were female. The sample varied in age, with very few participants over 65. The majority (95.6%) of the sample have education higher than a bachelor’s degree; 47% reported professional study as their highest degree, and 30.9% reported having a continuing education after their bachelor’s degree. The participants´ professions varied. The biggest group comprises clinical psychologists (30%) and clinical psychologist specialists (17.9%). The second largest group consists of social workers (15.5%), followed by nurses (12.6%) and psychiatrists (9.2%).

**Table 1:** Background characteristics of respondents (*N* = 207)

Sample characteristics	Percentage	Number per characteristic
**Gender**		
Female	84.1	174
Male	15.9	33
**Age**		
26–33	19.3	40
34–41	21.3	44
42–49	30.0	61
50–57	18.4	38
58–65	10.1	21
66–73	1.0	2
**Level of education**		
Bachelor’s degree	3.9	10
Bachelor’s degree with continuing education	30.9	62
Master’s degree	11.1	23
PhD degree	6.3	13
Professional study	47.3	98
**Job position**		
Clinical Psychologist	30.0	62
Clinical Psychologist Specialist	17.9	37
Nurse	12.6	26
Social Educator	2.6	5
Social Worker	15.5	32
Junior Medical Doctor	1.4	3
Psychiatrist	9.2	19
Other Health/Therapy Personnel with a higher education	6.3	2
Other	4.8	10
**Area of service**		
Specialized Mental Health Clinics	59.4	123
Specialized Drug Addiction and Treatment Clinics	5.8	12
Municipal Mental Health Services	26.6	52
Norwegian Labor and Welfare Administration	2.9	6
Other	6.3	14
**Total number of informants**		**207**

### Routines for integrating employment as a part of treatment for people with moderate to severe mental illness

As seen in Table 2, over half (58.1%) of the mental health professionals stated that employment is often discussed, 31.5% reported that it is discussed sometimes, while only 7.9% stated that it is never or rarely discussed. Results show that all areas of practice frequently engage in employment conversations at the respective unit. The majority of mental health professionals working in social services (80%) discuss employment opportunities, activity or collaboration, followed by specialized care, drug addiction and treatment clinics (72.7%), specialized care, mental health clinics (59.8%) and lastly, those working in municipal mental health services (53.8%).

**Table 2:** Efforts to include employment among mental health professionals (*N* = 207)

	Area of service	Other (*n* = 14)	Municipal Mental health Services (*n* = 52)	Social Services (*n* = 6)	Specialized Care, Mental Health Clinics (*n* = 123)	Specialized Care, Drug Addiction and Treatment Clinics (*n* = 12)	Total
How often are patients’ ability to work/employment opportunities, work-oriented activity or collaboration with social services a topic in staff meetings the unit?	Often	38.5%	53.8%	80%	59.8%	72.7%	58.1%
	Sometimes	38.5%	28.8%	20%	33.6%	18.2%	31.5%
	Seldom or never	15.4%	17.3%	0%	3.3%	9.1%	7.9%
	Do not know	7.7%	0%	0%	3.3%	0%	2.5%
	Total						100%
Does the unit have routines for mental health professionals to address work or work-oriented activity as a topic in consultations with patients (for example, conversation tools or treatment plan?	Yes	53%	49.1%	33.3%	66.7%	58.3%	59.9%
	No	23.1%	39.6%	0%	17.1%	16.7%	22.7%
	Do not know	23.1%	9.4%	50%	15.4%	16.7%	15.5%
	Total						100%

When asked about routines for addressing employment, over half of the mental health professionals reported yes (59.9%) and 15.5% did not know. In terms of area of practice, 66.7% of those working in specialized care, municipal mental health services reported that they have routines for addressing employment or work-related activities with patients, compared to 33.3% in social services, 58.3% in specialized care, drug addiction and treatment clinics, and 49.1% in municipal mental health services. Half of mental health professionals in social services (50%) did not know whether the unit have routines for mental health providers addressing employment or work-related activities with patients. In comparison, 9.4% of the mental health professionals in municipal mental health services did not know of employment routines, 15.4% of those working in specialized care mental health clinics, and 16.7% of those working in specialized care, drug addiction and treatment clinics did not know of routines for addressing employment with patients.

### Expectations about employment for people with moderate to severe mental illness

Table 3 shows the overall mean scores and standard deviations for each subscale and for EESMI. Results suggested global favorable expectations of employment for people with moderate to severe mental illness. In terms of each subscale, respondents reported the most positive attitudes about the benefits of work, followed by motivation to work, and lastly the demands of the worker role.

**Table 3:** Mean subscales and overall EESMI

Scale	M	SD
Benefits of work	5.39	.76
Motivation to work	5.34	.56
Demands of the worker role	4.55	.89
EESMI	5.12	.56

Expectations are measured on a 7-point Likert scale (strongly disagree-strongly agree)

### Expectations about employment and the participants profession

 To study variation within the sample, a one-way ANOVA and Tukey’s multiple comparisons was used to examine the relationships between the four largest professions (psychologist, nurse, social workers, and psychiatrists) and the three dependent variables, ‘benefits of work’, ‘demands of the worker role’ and ‘motivation to work’ (Table 4).

**Table 4:** Expectations about employment: multiple comparisons of professional groups

	Psychologist	Nurse	Social Worker
Psychologist (*n* = 94; *n* = 96)	-	-	-
(M Benifits = 54.0; SD = 7,7; SE = 0.8) (M Demands = 32.2; SD = 5.3; SE = 0.5) (M Motivation = 26.9; SD = 4.5; SE = 0.4)			
Nurse (*n* = 26; *n* = 26)	Benifits Mdiff = 1.05, *p* = 0.92 Demands Mdiff = –.91, *p* = 0.84 Motivation Mdiff = -1.67, *p* = 0.29	**-**	**-**
(M Benifits = 55.1; SD = 7.3; SE = 1.4) (M Demands 31.3; SD = 4.9; SE = 0.9) (M Motivation = 25.2; SD = 4.1; SE = 0.4)			
Social Worker (*n* = 30; *n* = 32)	Benifits Mdiff = 1.78, *p* = 0.81 Demands Mdiff = 0.35, *p* = 0.98 Motivation Mdiff = -1.48, *p* = 0.91	Benifits Mdiff = 1.17, *p* = 0.81 Demands Mdiff = -1.26, *p* = 0.79 *Motivation Mdiff = -3.16, *p* = 0.37	**-**
(M Benefits = 53.3; SD = 6.6; SE = 1.2) (M Demands = 32.6; SD = 4.6; SE = 0.8) (M Motivation = 28.4; SD = 3.1; SE = 0.6)			
Psychiatrist (*n* = 18; *n* = 17)	Benifits Mdiff = 0.60, *p* = 0.91 Demands Mdiff = 1.61, *p* = 0.65 Motivation Mdiff = -2.42, *p* = 0.16	Benifits Mdiff = -0.45, *p* = 0.99 Demands Mdiff = -0.69, *p* = 0.97 Motivation Mdiff = -0.74, *p* = 0.94	Benifits Mdiff = -0.33.17, *p* = 0.94 Demands Mdiff = -1.96, *p* = 0.61 *Motivation Mdiff = 3.91, *p* = 0.02
(M Benifits = 54.6; SD = 6.1; SE = 1.5) (M Demands = 30.6; SD = 3.8; SE = 0.9) (M Motivation = 24.5; SD = 3.3; SE = 0.8)			

*M* Mean, *SD* Standard deviation, *SE* Standard error, *M*_*diff*_ Mean difference

^*^statistically significant difference (*p* ˂ 0.05)

The analyses demonstrated that there are some significant differences between profession and the dependent variable ‘motivation to work’. Results demonstrated that nurses scored lower than social workers (Mdiff = -3.16, *p* = 0.03), and psychiatrists scored substantially lower than social workers (Mdiff = -3.91, *p* = 002), which suggests expectations about employment for people with moderate to severe mental illness can to some degree be attributed to the participants profession.

### Predictors of expectations toward employment for people with moderate to severe mental illness

Multiple linear regression was used to determine the relationship between the dependent variables ‘motivation to work’, ‘demands of the worker role’, ´benefits of work ‘and nine background variables. Professions with a low number of participants were omitted. The first model was significant: F (3.455) with an *R*^*2.*^value of 0.10, suggesting that the model explained 10% of the variation in the dependent variable. The background variables include gender, education, profession, tenure, patient participation in meetings, and interaction with social services.

As seen in Table 5, the results demonstrate that being a psychiatrist is negatively associated with the dependent variable ‘motivation to work’, which reflects expectations that individuals with moderate to severe mental illness are motivated to pursue and maintain employment. The variable “patient interaction” was positively associated with motivation to work, indicating that the patient attending meetings with external service providers when the focus of the meeting is about the patent, contribute to positive expectations about employment for people with moderate to severe mental illness. The second and third model with the predictors ‘demands of the worker role’ and ‘benefits of work’ were non-significant.

**Table 5:** Predictors of employment for people with moderate to severe illness (*n* = 199)

Variable	**Motivation to work**			**Demands of the worker role**			**Benefits of work**		
	*β*	SE	*p*	*β*	SE	*p*	*β*	SE	*p*
Gender (female)	0.93	.85	.184	-.050	1.04	.500	-.135	1.52	.069
Master’s degree (ref: bachelors and continuing education)	-.059	.982	.576	.091	1.17	.408	.005	1.78	.966
Psychologist (Clinical and Specialized)	.099	.924	.291	.065	1.10	.510	.111	1.64	.267
Nurse	-.157	1.18	.079	.019	1.41	.837	.068	2.17	.475
Social Worker	.091	1.07	.284	.097	1.28	.283	.016	1.95	.862
Psychiatrist	-.224**	1.18	.004	-.81	1.44	.310	-.045	2.09	.578
Tenure	-060	.720	.449	.011	0.85	.892	.037	1.28	.666
Patient participation	.140*	1.09	.046	-.066	1.29	.367	.037	1.94	.617
Interaction with Social Services	-.060	.64	.387	.141	0.76	.055	-.034	1.16	.645
*R*^*2*^	.100				-.005		-0.14		
F	3.455				.544		.717		

^**^p < 0.01; ^*^p < 0.05

## Discussion

The purpose of this study was to examine mental health professionals’ expectations and efforts to include employment for people with moderate to severe mental illness. The mean total of EESMI was 5.12 (*SD* = 0.56), suggesting global favorable expectations of employment for people with moderate to severe mental illness. Findings in this study are comparable to previous studies using EESMI which have shown moderate to high overall scores using EESMI (M = 1.8, SD = 0.4) among case managers [26] and community mental health center staff (M = 2.00, SD = 0.41) on a three-point scale [28]. However, some previous research on employment for people with mental illness demonstrates more negative expectations [19–21]. For example, Kochański et al. [19] found that a large group of psychiatrists in Poland hold stigmatizing attitudes towards people with mental illness and their ability to work. The qualitative study by Roets et al. [20] finds negative expectations about people with mental illness and their ability and competence to hold a job among mental health professionals, arguably attributed to stereotypes and prejudice about people with mental illness and their ability and competence to hold a job. Similarly, the study by Costa et al. found that providers in the U.S. rated employment and finances as the least important factors in promoting the recovery of people with mental illness. Here, the authors argue that this may be explained by the historical view that people with mental illness are unable to, and uninterested in working [21]. One possible explanation for the favorable expectations about employment in this study might be attributed to the upscaling of IPS in Norway the past decade, both through government funding and support from mental health professionals and academia [29], and the integration of employment specialists in specialized mental health services [30], which has resulted in an emphasis on integrating employment in treatment settings.

The main finding from the multiple linear regression analyses revealed that patients attending meetings with external service providers when the meeting is about him/herself predict positive expectations about the patients’ motivation to work, that is, individuals with moderate to severe mental illness’s motivation to pursue and maintain employment. Although user participation is a statutory right in Norwegian law, requirements are not always met [31]. The findings in this study reflect how user participation influence mental health professionals’ expectations about employment activation, hence underscoring the importance of upholding the requirement of user participation during treatment processes.

In addition, findings reveal significant differences between professional backgrounds and the dependent variable ‘motivation to work’, assessing expectations about whether individuals with moderate to severe mental illness are motivated to pursue and maintain employment. Results suggested that some psychiatrists hold negative expectations, particularly in comparison to social workers. One possible explanation for this finding may be the different educational focus between psychiatrists and social workers. For instance, social work as a professional generally has strong historical ties to labor activation and emphasizes the benefits of employment in social work education. This educational focus may be less apparent among psychiatrists. Findings may also reflect negative attitudes, skepticism about employment for people with mental illness, and the idea that patients must be symptom free before seeking out employment [16, 20, 32]. While surprising, these results must, however, be read with caution due to the low sample size among psychiatrists.

In terms of mental health professionals’ efforts to include employment for people with moderate to severe mental illness in their daily practice, results suggests that a little over half (58.1%) of mental health professionals have integrated discussions about patients’ ability to work/employment opportunities, work-oriented activity, or collaboration with social services. Of those, 53.3% working in municipal mental health services report that they often discuss it, compared to 80% in social services, 59% in specialized care mental health services and 72% in specialized care, drug addiction and treatment clinics, suggesting that those in social services more often discuss patients’ ability to work/employment opportunities compared to other areas of practice. Regarding whether their unit has routines for mental health professionals to address work or work-oriented activity as a topic in consultations with patients, 59.9% of the mental health professionals answered yes. Om the other hand, these results demonstrate that 37.7% of the participants stated that they do not or do not know whether their unit has routines for mental health professionals to address work or work-oriented activity as a topic in consultations with patients. These results reflect previous research underscoring fragmented collaboration agreements between social services and health trusts in Norway [25], and emphasize the importance of strengthening interprofessional collaborations. This suggests that we still have a long way to go before employment is fully integrated in the treatment practices of mental health professionals in Norway.

This study points to patient participation and professional background being important predictors in expectations about employment for people with mental illness. In addition, findings illustrate substantial variations in how practices surrounding employment is integrated in mental health services. However, more research is needed to examine the underlying mechanisms of employment inclusion in treatment settings in Norway. One way of doing this is to study the implementation of employment interventions in mental health contexts and assess barriers for integrating employment in treatment settings. A central aspect of this implementation is collaboration between mental health services and social services [33, 34]. Hence, future research should also examine collaborative employment practices between services to strengthen integrated mental health care for people with moderate to severe mental illness.

### Study limitations

There are some limitations in this study that need to be addressed. First, we used purposive snowball sampling to reach mental health providers in various fields of practice with different professional backgrounds across Norway. Although the sample is diverse, the snowball sampling makes difficult to determine representativeness and the sample. Due to the recruitment method, comparisons with the population are particularly challenging. One notable characteristics of the sample is that the majority were female (84.1%), which is not surprising considering about 82% of professionals employed in the health-and social sector in Norway are female [35]. In terms of demographic variables such as age and profession, there is no comparable population data to determine representativeness. Generalization of results is therefore difficult. Second, regression analyses can be negatively affected by insufficient sample sizes, thus reducing the statistical power of the study and margin of error. Specifically, the low number of participants represented in professional backgrounds and field of practice might have influenced the regression outcome.

## Data Availability

The data that support the findings of this study are available upon request of the first author.

## References

[1] OECD. Better life index - How's Life in Norway?. 2020. Available from: https://www.oecd.org/norway/Better-Life-Initiative-country-note-Norway.pdf.

[2] Steen Tesli M, Handal M, Ask Torvik F, Skrindo Knudsen AK, Odsbu I, Gustavson K (2016). Mental illness among adults Norwegian Institute of Public Health.

[3] Bartelink VHM, Zay Ya K, Guldbrandsson K, Bremberg S (2020). Unemployment among young people and mental health: A systematic review. Scand J Public Health.

[4] Brouwers EPM (2020). Social stigma is an underestimated contributing factor to unemployment in people with mental illness or mental health issues: Position paper and future directions. BMC Psychol.

[5] Leufstadius C, Eklund M, Erlandsson L-K (2009). Meaningfulness in work - Experiences among employed individuals with persistent mental illness. Work.

[6] Abraham KM, Chang M-UM, Van T, Resnick SG, Zivin K (2021). Employment After Vocational Rehabilitation Predicts Decreased Health Care Utilization in Veterans With Mental Health Diagnoses. Mil Med.

[7] Modini M, Joyce S, Mykletun A, Christensen H, Bryant RA, Mitchell PB (2016). The mental health benefits of employment: Results of a systematic meta-review. Australas Psychiatry.

[8] Sveinsdottir V, Bull HC, Evensen S, Reme SE, Knutzen T, Lystad JU (2020). A Short History of Individual Placement and Support in Norway. Psychiatr Rehabil J.

[9] Bond GR, Drake RE, Becker DR (2008). An Update on Randomized Controlled Trials of Evidence-Based Supported Employment. Psychiatr Rehabil J.

[10] Sveinsdottir V, Lie SA, Bond GR, Eriksen HR, Tveito TH, Grasdal AL (2020). Individual placement and support for young adults at risk of early work disability (the SEED trial). A randomized controlled trial. Scand J Work Environ Health.

[11] Frederick DE, VanderWeele TJ (2019). Supported employment: Meta-analysis and review of randomized controlled trials of individual placement and support. PLoS One.

[12] American Psychiatric Association. OECD Report Highlights Impact of Mental Illness on the Workforce, Recommends Policy Changes. Psychiatr Serv 2012;63(1):96. 10.1176/appi.ps.201200p96 22227770

[13] Gmitroski T, Bradley C, Heinemann L, Liu G, Blanchard P, Beck C (2018). Barriers and facilitators to employment for young adults with mental illness: a scoping review. BMJ Open.

[14] Bond GR, Drake RE, Becker DR (2020). An update on Individual Placement and Support. World Psychiatry.

[15] Bonfils IS, Hansen H, Dalum HS, Eplov LF (2017). Implementation of the individual placement and support approach – facilitators and barriers. SJDR.

[16] Khalifa N, Hadfield S, Thomson L, Talbot E, Bird Y, Schneider J (2020). Barriers and facilitators to the implementation of individual placement and support (IPS) for patients with offending histories in the community: The United Kingdom experience. BJOT.

[17] Biggs D, Hovey N, Tyson PJ, MacDonald S (2010). Employer and employment agency attitudes towards employing individuals with mental health needs. J Ment Health.

[18] Janssens KME, van Weeghel J, Dewa C, Henderson C, Mathijssen JJP, Joosen MCW (2021). Line managers’ hiring intentions regarding people with mental health problems: a cross-sectional study on workplace stigma. Occup Environ Med.

[19] Kochański A, Cechnicki A (2017). The attitudes of psychiatrists toward people suffering from mental illnesses. Psychiatr Pol.

[20] Roets G, Kristiansen K, Van Hove G, Vanderplasschen W (2007). Living through exposure to toxic psychiatric orthodoxies: exploring narratives of people with 'mental health problems' who are looking for employment on the open labour market. Disabil Soc.

[21] Costa M, Baker M, Davidson L, Giard J, Guillorn L, Ibáñez AG (2017). Provider perspectives on employment for people with serious mental illness. Int J Soc Psychiatry.

[22] Gowdy EA, Carlson LS, Rapp CA (2004). Organizational Factors Differentiating High Performing from Low Performing Supported Employment Programs. Psychiatr Rehabil J.

[23] Sackett DL (2000). Evidence-based medicine : how to practice and teach EBM.

[24] Ruud T, Friis S (2021). Community-based Mental Health Services in Norway. Consortium Psychiatricum.

[25] Proba Samfunnsanalyse. Arbeidsfokus på DPS – samarbeid med NAV [Work focus at DPS - Collaboration with NAV]. PROBA. 2016. Available from https://proba.no/wp-content/uploads/proba-rapport-2016-03-arbeidsfokus-pa-dps--samarbeid-med-nav.pdf.

[26] Abraham KM, Stein CH (2009). Case Managers' Expectations about Employment for People with Psychiatric Disabilities. Psychiatr Rehabil J.

[27] Sousa VD, Rojjanasrirat W (2011). Translation, adaptation and validation of instruments or scales for use in cross-cultural health care research: a clear and user-friendly guideline. J Eval Clin Pract.

[28] Brucker DL, Doty M (2019). Community Mental Health Center Staff Attitudes About Employment for Persons With Serious Mental Illness. Psychiatr Rehabil J.

[29] Fyhn T, Øygarden O, Monstad K, Skagseth M. Evaluering av samarbeidet mellom NAV og helsetjenesten om Individuell jobbstøtte (IPS). [Evaluation of the collaboration between NAV and the health service on individual placement and support). NORCE Norwegian Research Centre AS 2021.

[30] European Union of Supported Employment (2010). European Union of supported employment toolkit.

[31] Skjærpe JN, Kristoffersen M, Storm M (2020). Service user involvement in mental healthcare coordination. Sykepleien forskning (Oslo).

[32] Bonfils IS, Hansen H, Dalum HS, Eplov LF (2017). Implementation of the individual placement and support approach–facilitators and barriers. SJDR.

[33] Moe C, Brinchmann B, Rasmussen L, Brandseth OL, McDaid D, Killackey E (2021). Implementing individual placement and support (IPS): the experiences of employment specialists in the early implementation phase of IPS in Northern Norway. The IPSNOR study. BMC Psychiatry.

[34] Spjelkavik Ø (2012). Supported Employment in Norway and in the other Nordic countries. J Vocat Rehabil.

[35] Statistics Norway (2022). Employees in the health- and social sector.

